# Supplementary material for: The OvarianTag™ Biomarker Panel Emerges as a Prognostic Tool to Guide Clinical Decisions in Cisplatin-Based Treatment of Epithelial Ovarian Cancer
Source: Int J Mol Sci. 2025 Aug 29;26(17):8393. doi: 10.3390/ijms26178393 (PMC12428831; doi:10.3390/ijms26178393)
Supplement: Supplementary file 1 [file ijms-26-08393-s001.zip › ijms-3508707-supplementary.pdf]

Supplementary material

Supplementary material Table S1: Candidate reference genes for normalization of qRT-PCR assays in studies of the expression profile of normal and tumor ovarian tissue

Case	18S	ALAS	GAPDH	SDHA	TBP	TFRC	Normalization Factor
70	0,0469	85,7	49,2	0,502	1,19	4,35	8,2482
63	0,217	40,7	33,5	0,00775	0,0337	20100	2,1383
68	0,00447	0,0141	0,0711	0,00184	0,0018	3,63	0,0567
**M < 0,15**	**4,684**	**4,24**	**3,608**	**3,464**	**3,303**	**8,523**	

The candidate reference genes were listed according to their expression stability (M value), calculated using the GeNorm algorithm. Genes highlighted in green represent the most stable, while the gene highlighted in red represents the least stable. The normalization factor was determined based on the geometric mean of the selected reference genes for each case.

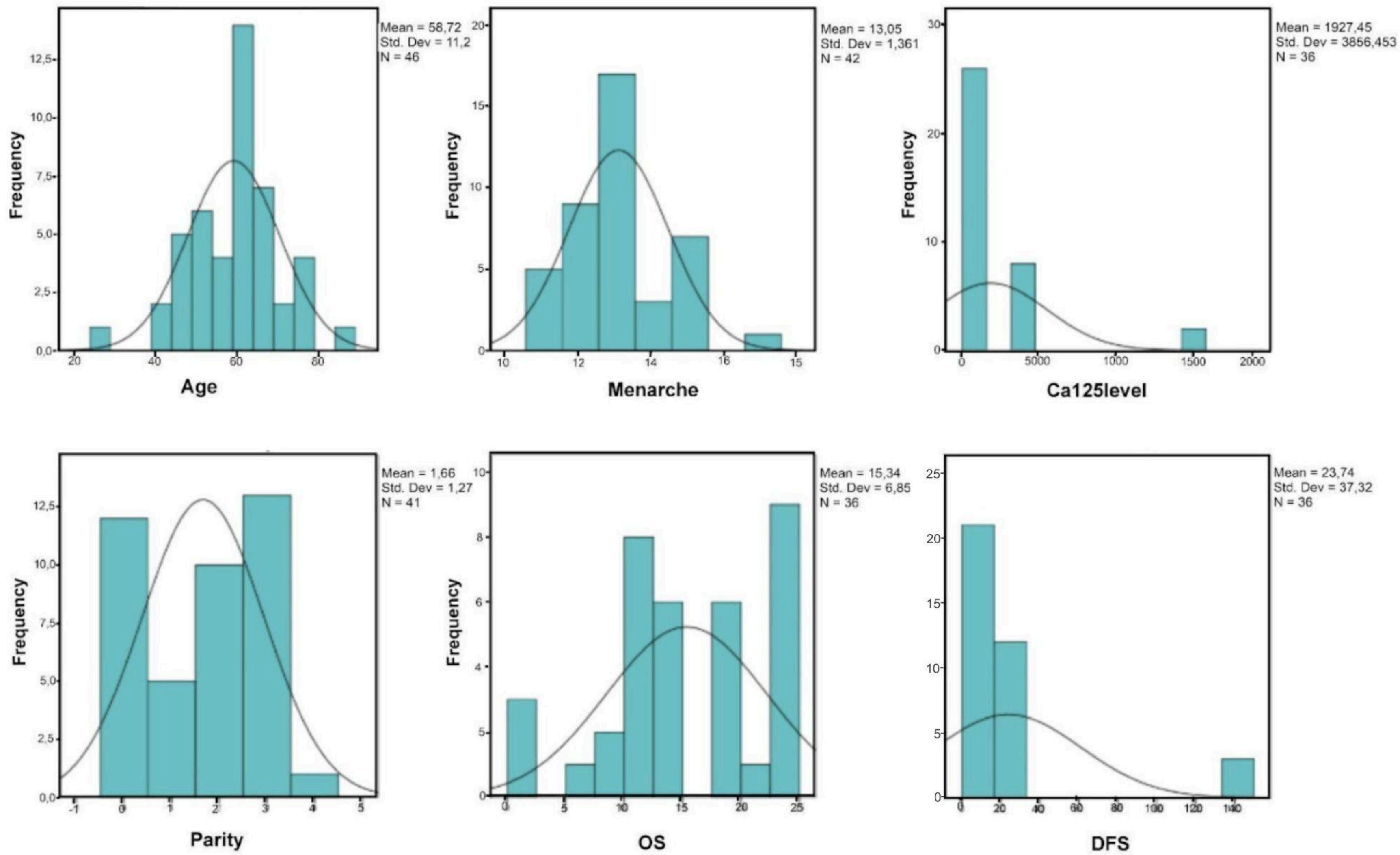

Supplementary material Figure S1: Frequency distribution of the clinical and pathological sample characteristics.

Supplementary material Table S2: Performance of individual attributes used in the classification models under full dataset training and Leave-One-Out Cross-Validation (LOOCV)

Compositions	FULL	LOOCV
2018_05_03_Oncotag_stage_NFKB_end	68% (17/25)	68% (17/25)
2018_05_03_Oncotag_stage_RIP_end	68% (17/25)	68% (17/25)
2018_05_03_Oncotag_stage_TANK_end	84% (21/25)	76% (19/25)
2018_05_03_Oncotag_stage_TNFRSF10D_end	68% (17/25)	44% (11/25)
2018_05_03_Oncotag_stage_TRAF2_end	68% (17/25)	68% (17/25)
2018_05_03_Oncotag_Ascites_NFKB_end	53.3333% (16/30)	43.3333% (13/30)
2018_05_03_Oncotag_Ascites_RIP_end	53.3333% (16/30)	53.3333% (16/30)
2018_05_03_Oncotag_Ascites_TANK_end	53.3333% (16/30)	30% (9/30)
2018_05_03_Oncotag_Ascites_TNFRSF10D_end	53.3333% (16/30)	53.3333% (16/30)
2018_05_03_Oncotag_Ascites_TRAF2_end	53.3333% (16/30)	53.3333% (16/30)
2018_05_03_Oncotag_CA-125_NFKB_end	75% (24/32)	75% (24/32)
2018_05_03_Oncotag_CA-125_RIP_end	75% (24/32)	75% (24/32)
2018_05_03_Oncotag_CA-125_TANK_end	93.75% (30/32)	84.375% (27/32)
2018_05_03_Oncotag_CA-125_TNFRSF10D_end	75% (24/32)	75% (24/32)
2018_05_03_Oncotag_CA-125_TRAF2_end	75% (24/32)	75% (24/32)
2018_05_03_Oncotag_ascites_genesapoptosis_end	53.3333% (16/30)	30% (9/30)
2018_05_03_Oncotag_CA-125_genesappoptose_end	96.875% (31/32)	81.25% (26/32)
2018_05_03_Oncotag_cytoreduction_genesapoptosis_end	56% (14/25)	52% (13/25)
2018_05_03_Oncotag_histology_genesapoptosis_end	50% (16/32)	3.125% (1/32)
2018_05_03_Oncotag_cytoreduction_NFKB_end	56% (14/25)	56% (14/25)
2018_05_03_Oncotag_cytoreduction_RIP_end	56% (14/25)	56% (14/25)
2018_05_03_Oncotag_cytoreduction_TANK_end	56% (14/25)	56% (14/25)
2018_05_03_Oncotag_cytoreduction_TNFSF10D_end	56% (14/25)	56% (14/25)
2018_05_03_Oncotag_cytoreduction_allgenes_end	72% (18/25)	60% (15/25)
2018_05_03_Oncotag_cytoreduction_TRAF2_end	56% (14/25)	52% (13/25)
2018_05_03_Oncotag_Degree_NFKB_end	68% (17/25)	56% (14/25)
2018_05_03_Oncotag_Degree_RIP_end	68% (17/25)	68% (17/25)
2018_05_03_Oncotag_Degree_TANK_end	68% (17/25)	68% (17/25)
2018_05_03_Oncotag_Degree_TNFRSF10D_end	68% (17/25)	44% (11/25)
2018_05_03_Oncotag_Degree_genesapoptosis_end	92% (23/25)	36% (9/25)
2018_05_03_Oncotag_histology_NFKB_end	43.75% (14/32)	43.75% (14/32)
2018_05_03_Oncotag_histology_RIP_end	43.75% (14/32)	34.375% (11/32)
2018_05_03_Oncotag_histology_TNFRSF10D_end	50% (16/32)	15.625% (5/32)
2018_05_03_Oncotag_histology_TRAF2_end	50% (16/32)	18.75% (6/32)
2018_05_03_Oncotag_OS_NFKB_end	56.25% (18/32)	56.25% (18/32)

2018_05_03_Oncotag_OS_RIP_end	56.25% (18/32)	56.25% (18/32)
2018_05_03_Oncotag_OS_TANK_end	62.5% (20/32)	53.125% (17/32)
2018_05_03_Oncotag_OS_TNFRSF10D_end	56.25% (18/32)	56.25% (18/32)
2018_05_03_Oncotag_OS_allgenes_end	62.5% (20/32)	50% (16/32)
2018_05_03_Oncotag_OS_TRAF2_end	56.25% (18/32)	53.125% (17/32)
2018_05_03_Oncotag_DFS_genesapoptosis_end	87.5% (28/32)	87.5% (28/32)
2018_05_03_Oncotag_OS_apoptosis_end	62.5% (20/32)	53.125% (17/32)
2018_05_03_Oncotag_DFS_NFKB_end	87.5% (28/32)	87.5% (28/32)
2018_05_03_Oncotag_DFS_RIP_end	87.5% (28/32)	87.5% (28/32)
2018_05_03_Oncotag_DFS_TANK_end	87.5% (28/32)	87.5% (28/32)
2018_05_03_Oncotag_DFS_TNFRSF10D_end	87.5% (28/32)	87.5% (28/32)
2018_05_03_Oncotag_DFS_allgenes_end	87.5% (28/32)	87.5% (28/32)
2018_05_03_Oncotag_DFS_TRAF2_end	87.5% (28/32)	87.5% (28/32)
2018_05_03_Oncotag_allgenes_ascites_end	53.3333% (16/30)	36.6667% (11/30)
2018_05_03_Oncotag_allgenes_CA-125_end	93.75% (30/32)	68.75% (22/32)
2018_05_03_Oncotag_allgenes_degree_end	84% (21/25)	68% (17/25)
2018_05_03_Oncotag_allgenes_histology_end	90.625% (29/32)	34.375% (11/32)
2018_05_03_Oncotag_allgenes_stage_end	100% (25/25)	40% (10/25)
2018_05_03_Oncotag_histology_TANK_end	43.75% (14/32)	34.375% (11/32)
2018_05_03_Oncotag_stage_genesapoptosis_end	96% (24/25)	64% (16/25)
